# Current EU regulatory requirements for the assessment of chemicals and cosmetic products: challenges and opportunities for introducing new approach methodologies

**DOI:** 10.1007/s00204-021-03034-y

**Published:** 2021-04-13

**Authors:** Francesca Pistollato, Federica Madia, Raffaella Corvi, Sharon Munn, Elise Grignard, Alicia Paini, Andrew Worth, Anna Bal-Price, Pilar Prieto, Silvia Casati, Elisabet Berggren, Stephanie K Bopp, Valérie Zuang

**Affiliations:** Directorate F–Health, Consumers and Reference Materials, Unit F3 Chemicals Safety and Alternative Methods, European Commission, Joint Research Centre (JRC), Via E. Fermi, 2749. TP126, 21027 Ispra, VA Italy

**Keywords:** EU regulatory requirements, Industrial chemicals, Cosmetic products, Cosmetic ingredients, Human health, Alternatives to animal testing, 3Rs, REACH, Cosmetic products regulation

## Abstract

The EU Directive 2010/63/EU   on the protection of animals used for scientific purposes and other EU regulations, such as REACH and the Cosmetic Products Regulation advocate for a change in the way toxicity testing is conducted. Whilst the Cosmetic Products Regulation bans animal testing altogether, REACH aims for a progressive shift from in vivo testing towards quantitative in vitro and computational approaches. Several endpoints can already be addressed using non-animal approaches including skin corrosion and irritation, serious eye damage and irritation, skin sensitisation, and mutagenicity and genotoxicity. However, for systemic effects such as acute toxicity, repeated dose toxicity and reproductive and developmental toxicity, evaluation of chemicals under REACH still heavily relies on animal tests. Here we summarise current EU regulatory requirements for the human health assessment of chemicals under REACH and the Cosmetic Products Regulation, considering the more critical endpoints and identifying the main challenges in introducing alternative methods into regulatory testing practice. This supports a recent initiative taken by the International Cooperation on Alternative Test Methods (ICATM) to summarise current regulatory requirements specific for the assessment of chemicals and cosmetic products for several human health-related endpoints, with the aim of comparing different jurisdictions and coordinating the promotion and ultimately the implementation of non-animal approaches worldwide. Recent initiatives undertaken at European level to promote the 3Rs and the use of alternative methods in current regulatory practice are also discussed.

## Introduction

The EU Directive 2010/63/EU (2010) on the protection of animals used for scientific purposes, other pieces of EU legislation, such as Regulation (EC) No 1907/2006 concerning the Registration, Evaluation, Authorisation and Restriction of Chemicals [REACH (2020g)] and the Regulation (EC) No 1223/2009 on cosmetic products (2020e), advocate for a change in the way toxicity testing is conducted, proposing a shift from in vivo testing, towards non-animal approaches based on in vitro and computational methods. This is considered essential to gather a deeper mechanistic understanding of chemical effects, taking into account human biology, and limiting (or avoiding) concerns associated with responses in test animals and humans due to interspecies differences.

At the European level, the need to integrate up-to-date in vitro and in silico methods and models in existing or new regulatory testing strategies has been promoted in Directive 2010/63/EU (2010), which includes a number of duties aimed at fostering the Replacement, Reduction and Refinement (i.e., Three Rs^1^) of animal testing. Additionally, REACH (2020g) and the Cosmetic Products Regulation (2020e) have either contributed to the implementation of the 3Rs by, respectively referring to and encouraging the use of alternatives to animal testing, or banning animal testing altogether. Along this line, for some specific toxicological endpoints (e.g., skin corrosion and irritation, serious eye damage and irritation, skin sensitisation, and mutagenicity and genotoxicity), the potential hazard of chemicals is often evaluated using non-animal approaches. Nevertheless, for other endpoints, such as acute systemic toxicity, repeated dose toxicity and reproductive and developmental toxicity, the regulatory requirements, and thus chemical safety evaluation, still heavily relies on the use of animals.

Understanding current regulatory requirements specific for the assessment of chemical and cosmetic ingredient effects on human health is essential to identify possible knowledge gaps, and evaluate how alternative (non-animal) methods could be integrated in current regulatory practice. This is in line with recent initiatives taken by the International Cooperation on Alternative Test Methods (ICATM) (https://ec.europa.eu/jrc/en/eurl/ecvam/alternative-methods-toxicity-testing/advisory-bodies/icatm), whose members include EURL ECVAM (European Union Reference Laboratory for Alternatives to Animal Testing) of the European Commission’s Joint Research Centre (JRC), ICCVAM (the US Interagency Coordinating Committee on the Validation of Alternative Methods) at the National Institute of Environmental Health Sciences, JaCVAM (Japanese Center for the Validation of Alternative Methods) at the National Institute of Health Sciences, Health Canada, and KoCVAM (South Korean Center for the Validation of Alternative Methods) at the National Institute of Environmental Health Sciences, with ad hoc participation from governmental institutions from Brazil, Singapore, China and Taiwan.

With the aim of comparing requirements in different jurisdictions and coordinating the promotion and ultimately the implementation of non-animal approaches worldwide, a summary of regulatory requirements for skin sensitisation testing across the countries represented by the ICATM partners was published (Daniel et al. 2018), together with a proposal of practical strategies to promote the adoption and regulatory use of defined approaches (DAs)^2^ for the assessment of skin sensitisation (Casati et al. 2018).

Here we summarise current EU regulatory requirements for the human health assessment of chemicals under REACH and the Cosmetic Products Regulation, considering the following toxicological endpoints: skin corrosion and irritation, serious eye damage/eye irritation, photo-induced toxicity, mutagenicity/genotoxicity, acute toxicity, skin sensitisation, repeated dose toxicity, carcinogenicity, reproductive and developmental toxicity, as well as absorption, distribution, metabolism and excretion (ADME) and toxicokinetics (TK), and identify the main challenges in current regulatory testing practice. We widen the discussion on the availability and advancement of new technologies and in vitro (non-animal) models, highlighting how new frameworks and initiatives undertaken at the European and international level could help to promote the 3Rs and implement twenty-first century test methods (NRC 2007) in current regulatory practice. Embracing a perspective that goes beyond specific regulatory silos and fostering knowledge sharing are essential to tackle complex human health-related endpoints.

## Current EU regulatory requirements of relevance for the safety assessment of chemicals and cosmetic products

Several pieces of EU Regulations and Guidance Documents (GDs) relevant for the safety assessment of industrial chemicals and cosmetic products are in place (as summarized in Table 1), which describe the information needed to assess potential environmental and human health-related adverse effects of industrial chemicals and cosmetic products.

**Table 1:** EU Regulations and guidance documents of relevance for the safety assessment of industrial chemicals and cosmetic products

EU Regulation or Guidance document	Content	Available at
**Regulation (EC) No 1272/2008** concerning the Classification Labelling and Packaging (CLP) of the European Parliament and of the Council of 16 December 2008 on classification, labelling and packaging of substances and mixtures	It ensures that the hazards presented by chemicals are clearly communicated to workers and consumers in the European Union through appropriate hazard symbols (pictograms) and labelling phrases. The need for risk assessment under REACH, a marketing ban, an authorization procedure for CMR substances, non-acceptance of chemicals in toys, cosmetics, storage of chemicals at industrial sites (Seveso Directive), the marketing to non-EU countries (Rotterdam convention), the definition of hazardous waste, the use of Eco-labels, etc. represent just some of the possible legal downstream consequences of CLP Regulation It aligns previous EU legislation on classification, packaging and labelling (Dangerous Substance Directive 67/548/EEC) of chemicals with the GHS (Globally Harmonized System) for Classification and Labelling (C&L) of Chemicals. While a manufacturer, importer or downstream user of any substance or mixture should not be obliged to generate new toxicological or eco-toxicological data for the purpose of classification, he should identify all relevant information available to him on the hazards of the substance or mixture and evaluate its quality. The manufacturer, importer or downstream user should also take into account historical human data, such as epidemiological studies on exposed populations, accidental or occupational exposure and effect data, and clinical studies. That information should be compared with the criteria for the different hazard classes and differentiations in order for that manufacturer, importer or downstream user to arrive at a conclusion as to whether or not the substance or mixture should be classified as hazardous Additional information regarding the application of CLP criteria can be found in (ECHA 2017b)	https://eur-lex.europa.eu/legal-content/EN/TXT/?uri=CELEX:02008R1272-20200501
**Regulation (EC) No 1907/2006** of the European Parliament and the Council of 18 December 2006 concerning the Registration, Evaluation, Authorisation and Restriction of Chemicals (REACH), establishing a European Chemicals Agency (ECHA)	The standard information requirements for the described endpoints are tonnage triggered (number of tonnes/year, tpy). This requires all companies manufacturing or placing a substance on the EU market in quantities greater than 1 tpy to register that substance with ECHA including cosmetic ingredients. The information required is dependent on the quantities (tonnage band) of a substance manufactured or imported within EU. In particular: – Standard information requirements for substances manufactured or imported in quantities of 1 tpy are provided in Annex VII; – Standard information requirements for substances manufactured or imported in quantities of 10 tpy or more are provided in Annex VIII; – Standard information requirements for substances manufactured or imported in quantities of 100 tpy or more are provided in Annex IX; –Standard information requirements for substances manufactured or imported in quantities of 1000 tpy or more are provided in Annex X; –General rules for adaptation of the standard testing regime set out in annexes VII to X are provided in Annex XI	https://eur-lex.europa.eu/legal-content/EN/TXT/?uri=CELEX:02006R1907-20200428
**ECHA Guidance** on Information Requirements and Chemical Safety Assessment, Chapter R.7a: Endpoint specific guidance Version 6.0	It describes the information requirements under REACH with regard to substance properties, exposure, uses and risk management measures, and the chemical safety assessment. It aims to help all stakeholders with their preparation for fulfilling their obligations under the REACH Regulation It highlights that, as per Annex VI, registrants should gather and evaluate all existing available information before considering further testing, such as physico-chemical properties, (Q)SAR, grouping, in vitro data, animal studies, and human data. For classified substances, information on exposure, use and risk management measures should also be collected and evaluated to ensure safe use of the substance. In case these data are inadequate for hazard and risk assessment, further testing should be carried out in accordance with the requirements of Annexes VII and VIII of REACH	https://echa.europa.eu/documents/10162/13632/information_requirements_r7a_en.pdf
**Regulation (EC) No 440/2008** of 30 May 2008 laying down test methods pursuant to Regulation (EC) No 1907/2006 of the European Parliament and of the Council on the Registration, Evaluation, Authorisation and Restriction of Chemicals (REACH)	The Regulation of May 2008 and its subsequent amendments (EC 2014) define which test methods are adopted to generate information on intrinsic properties of substances for the REACH Regulation. They are mostly based on the OECD Test Guidelines (TGs) for the Testing of Chemicals. In an upcoming amendment, it is foreseen that methods will not be any longer annexed, and only references to OECD TGs will be provided, unless no OECD TG is available or the test method and its respective TG are still aligned	https://eur-lex.europa.eu/legal-content/EN/TXT/?uri=CELEX:02008R0440-20191016
**Regulation (EC) No 1223/2009** of the European Parliament and the Council of 30 November 2009 on cosmetic products	It establishes rules to be complied with by any cosmetic product made available on the market, to ensure the functioning of the internal market and a high level of protection of human health. Animal testing has not been allowed for cosmetics and their ingredients since 11 March 2013 for any toxicological endpoint, due to a testing and marketing ban taken up in the 7th Amendment of the Cosmetics Directive [2003/15/EC]. For cosmetic products and their ingredients, scientifically valid alternative methods have to be applied to evaluate their safety. The Regulation prohibits (article 18) the placing on the market of: –cosmetic products where the final formulation has been the subject of animal testing; –cosmetic products containing ingredients or combinations of ingredients which have been the subject of animal testing Without prejudice to the above, the toxicological profile of all substances contained in the cosmetic product should be made for all relevant toxicological endpoints. A particular focus on local toxicity evaluation (skin and eye irritation), skin sensitisation, and, in the case of UV absorption, photo-induced toxicity is necessary All significant toxicological routes of absorption should be considered as well as the systemic effects and margin of safety (MoS) based on a no observed adverse effects level (NOAEL) should be calculated. The absence of these considerations should be duly justified	https://eur-lex.europa.eu/legal-content/EN/TXT/?uri=CELEX:02009R1223-20200501
**SCCS/1602/18** Scientific Committee on Consumer Safety (SCCS) Notes of Guidance (NoG) for the Testing of Cosmetic Ingredients and their Safety Evaluation, 10th revision	It contains relevant information on the different aspects of testing and safety evaluation of cosmetic substances listed in the annexes of the Cosmetic Products Regulation in Europe. The emphasis of this guidance is on cosmetic ingredients, although some guidance is also given for the safety assessment of finished products in previous versions. In the EU, the safety of cosmetic products is based on the safety of the ingredients, the rationale for this coming from the fact that many thousands of different cosmetic products on the EU market are all derived from a limited number of substances. Thus, toxicity testing has been concentrated on ingredients, and particularly on those that are intended to react with biological systems and therefore are of potential concern for human health. This is also the basis for the lists of authorised and banned and restricted substances in the Annexes I-VI of the Cosmetic Products Regulation. The Notes of Guidance (NoG) are designed to provide guidance to public authorities and to the cosmetic industry to improve harmonised compliance with the Cosmetic Products Regulation. Since the EU cosmetic legislation prohibits the marketing of finished products containing ingredients or combinations of ingredients that have been subject to animal testing after 2013 (see above), the SCCS has closely followed the progress made with regard to the development and validation of alternative methods For the safety evaluation of cosmetic ingredients two channels are functional. The safety of the Annex substances is evaluated by the SCCS; the safety of cosmetic products with all their ingredients is evaluated by the industry placing them on the EU market. Thus, the Annex substances fall under the responsibility of the SCCS Guidance on how to comply with the testing bans is given in the SCCS Notes of Guidance (SCCS 2018), and a factsheet has also been published by ECHA with respect to the interface between REACH and Cosmetic Products Regulations (ECHA 2014a) (Section 3-1 of the NoG)	https://ec.europa.eu/health/sites/health/files/scientific_committees/consumer_safety/docs/sccs_o_224.pdf

### Interface between REACH and the Cosmetic Products Regulation

A joint ECHA-Commission statement (ECHA 2014a) clarified the interface between REACH and the Cosmetic Products Regulation. According to that statement, the animal testing ban in the Cosmetic Products Regulation concerns the tests needed to prove safety of the cosmetic products on the ‘end users’ (e.g., consumers). The marketing ban of cosmetic products that have been tested on animals is triggered, if the results of a study on vertebrate animals, required pursuant to the information requirements set out in the REACH Regulation, are relied on in the cosmetic product safety report under the Cosmetic Products Regulation to demonstrate the safety for the end user of products containing the registered substance exclusively used in cosmetic products.^3^

However, the risks arising from other sources of exposure than the end use of cosmetic products are not assessed under the Cosmetic Products Regulation. In particular, REACH requires the evaluation of the risks to workers and the environment if the substance is covered by this Regulation. Regarding the relationship between REACH and the Cosmetic Products Regulation, decisions of the board of appeal of ECHA have been taken and are currently challenged by NGOs and Cosmetics industry, as reported in case numbers A-009-2018^4^ and A-010-2018,^5^ which consider examples of substances exclusively used as an ingredient in cosmetic products.

Even if a substance is registered exclusively for cosmetic use, REACH animal testing requirements continue to apply, as a last resort, to assess the risk from exposure to workers and for all environmental endpoints. REACH does not contain an automatic exemption from the information requirements for registration if an ingredient is used as a substance in cosmetic products only. A registrant can benefit from an exemption only if he/she shows that the conditions for an adaptation (e.g., a waiver for the studies) are fulfilled. The animal testing ban under the Cosmetic Products Regulation, therefore does not prevent registrants from carrying out tests to comply with the information requirements of REACH. It is important to note though that tests on vertebrate animals should only be carried out as a very last resort, i.e., when no information which meets the information requirements is already available, and where no adaptation (e.g., where no alternative tests exist) can be applied. Moreover, it should also be noted that only the Court can provide a legally binding interpretation of Union law and more particularly on the relationship between the testing and marketing bans in the Cosmetic Products Regulation and the requirements of the REACH Regulation.

This manuscript focuses in particular on the regulatory requirements for relevant human-health related endpoints. More detailed information regarding these endpoints is reported in the next sections.

### Skin corrosion and irritation and serious eye damage/eye irritation

Under CLP Regulation (2020f), the criteria for skin corrosive category and subcategories and skin irritation category are based on animal data; however, validated and accepted in vitro alternatives may also be used to help make classification decisions. The criteria for skin corrosion and irritation was updated to include criteria for the application of non-animal methods in the 8th revision of the GHS (UN-GHS 2019), and the CLP Regulation implementing GHS within the EU, will be revised accordingly.

Category 1 applies to corrosive substances, which can be further divided into three subcategories: category 1A, 1B and 1C, applied in the GHS and Packing Groups I, II and III applied in the UN Model Regulations for transport of dangerous goods. In the 21st revision of the Model Regulations (UN-TDG 2019) and in the 8th revision of GHS (UN-GHS 2019) the possibility for sub-classification based on in vitro data was introduced. Category 2 is attributed to irritant substances. Category 3 (mild skin irritation) is optional and is available for those authorities that want more than one skin irritation category (e.g., for classifying pesticides).

OECD GD 237 (OECD 2016a) describes waiving principles applicable to mammalian acute toxicity (oral, dermal and inhalation route), eye and skin irritation and skin sensitisation, intended for pesticides, but extendable also to other chemicals, formulations and biological materials. As specified in the OECD GD 237, “*In the context of this document, acute toxicity studies refer to those assessing systemic toxicity as well as those assessing local irritation, corrosion or sensitisation*”.

The Classification and Labelling (C and L) categories used are based on visually observable effects in rabbit skin following Draize skin corrosion and skin irritation test [EU test method B.4, equivalent to OECD TG 404 (OECD 2015c)]. However, as for skin corrosion/irritation, validated and accepted in vitro alternatives shall be used to make classification decisions (EC 2017d). This is also confirmed in the GHS Fig. 3.2.1, which reports tiered testing and evaluation of skin corrosion and irritation potential (see line 28f).

For serious eye damage/eye irritation, the classification system involves a tiered testing and evaluation scheme. The criteria themselves for irreversible or reversible eye effects are still based on animal data. On GHS level, the criteria for serious eye damage and eye irritation is currently under revision, and an updated text to include non-animal criteria is expected in the 9th revision of GHS in 2021. A substance or mixture classified as corrosive to skin is deemed to be classified for serious eye damage, to avoid any testing of corrosive substances for eye effects in vivo (ECHA 2017c).

Under REACH (2020g), for Annex VII and Annex VIII the assessment of skin irritation or skin corrosion using an in vitro test is foreseen. Regarding serious eye damage/eye irritation, the basic information requirement is an in vitro study, and a second in vitro study must be considered if the results from the first in vitro study do not allow a conclusive decision on classification for serious eye damage/eye irritation. Annex VIII foresees the assessment of skin irritation using the in vivo test only if the in vitro studies (under Points 8.1.1 and 8.1.2 of Annex VII) are not applicable, or their result(s) not adequate for classification and risk assessment. Same consideration is made for eye irritation. These amendments to Annexes VII and VIII relevant for skin corrosion/irritation and serious eye damage/eye irritation have been made in 2016 (EC 2016), considering the significant scientific progress in the development of alternative test methods for these endpoints. In particular, for both skin corrosion/skin irritation and serious eye damage/eye irritation, adequate information for the classification and risk assessment of a substance should be obtained in most cases solely on the basis of in vitro studies. For both these endpoints, in vivo studies may still be required in some cases for substances manufactured or imported in quantities of 10 tpy or more. Therefore, Points 8.1 and 8.2 of Annex VIII were amended so that the standard information requirements are now for the in vitro studies, while setting the conditions under which an in vivo study for skin irritation/corrosion and serious eye damage/eye irritation is still required.

Adopted in vitro OECD TGs and corresponding test methods indicated in Regulation 440/2008 (2019b) for skin corrosion/irritation and serious eye damage/eye irritation are reported in Table 2.

**Table 2:** Currently available Test Methods in Regulation (EC) No 440/2008 and corresponding OECD Test Guidelines (TGs)

Human health endpoint	Test methods/OECD TGs	In vivo/ in vitro
Skin corrosion/irritation	B.46. In vitro skin irritation: reconstructed human epidermis (RhE) test method [equivalent to OECD TG 439 (OECD 2015f)]	In vitro
	B.40. In vitro skin corrosion: transcutaneous electrical resistance test (TER) [equivalent to OECD TG 430 (OECD 2015d)]	In vitro
	B.40 Bis. In vitro skin corrosion: Human skin model test [equivalent to OECD TG 431 (OECD 2016g)]	In vitro
	In vitro Membrane Barrier Test Method (OECD TG 435) (OECD 2015e)	In vitro
	B.4: Acute Dermal Irritation/Corrosion [equivalent to OECD TG 404 (OECD 2015c)]	In vivo
Serious eye damage/irritation	B.47. Bovine Cornea Opacity Permeability (BCOP) Test Method [equivalent to OECD TG 437 (OECD 2017e)]	In vitro
	B.48. Isolated Chicken Eye (ICE) test method [equivalent to OECD TG 438 (OECD 2018h)]	In vitro
	B.61. Fluorescein Leakage (FL) Test Method [equivalent to OECD TG 460 (OECD 2017f)]	In vitro
	Short Time Exposure (STE) Test Method (OECD TG 491) (OECD 2018p)	In vitro
	Reconstructed human Cornea-like Epithelium (RhCE) Test Method (OECD TG 492) (OECD 2018q)	In vitro
	Vitrigel-Eye Irritancy Test Method (OECD TG 494) (OECD 2019a)	In vitro
	In vitro Macromolecular Test Method (OECD TG 496) (OECD 2019b)	In vitro
	B.5: Acute eye irritation/corrosion (equivalent to OECD TG 405 (OECD 2017d))	In vivo
Photo-induced toxicity	B.41 In vitro 3T3 NRU Phototoxicity Test [equivalent to OECD TG 432 (OECD 2004c)]	In vitro
Mutagenicity/Genotoxicity	B.10. Mutagenicity – In vitro Mammalian chromosome aberration test [equivalent to OECD TG 473 (OECD 2016i)]	In vitro
	B.11. Mutagenicity—In vivo Mammalian bone marrow chromosome aberration test [equivalent to OECD TG 475 (OECD 2016k)]	In vivo
	B.12. Mutagenicity—In vivo Mammalian erythrocyte micronucleus test [equivalent to OECD TG 474 (OECD 2016j)]	In vivo
	B.13/14. Mutagenicity: Reverse mutation test using bacteria [equivalent to OECD TG 471 (OECD 1997b)]	In vitro
	B.17. Mutagenicity—In vitro Mammalian cell gene mutation test [equivalent to OECD TG 476, which has been recently updated and split into TG 476 (OECD 2016l ) and TG 490 (OECD 2016q)]	In vitro
	B.22. Rodent dominant lethal test [equivalent to OECD TG 478 (OECD 2016m)]	In vivo
	B.23. Mammalian Spermatogonial Chromosome aberration test [equivalent to OECD TG 483 (OECD 2016n)]	In vivo
	B.25. Mouse Heritable Translocation [equivalent to OECD TG 485 (OECD 1986), although almost never requested]	In vivo
	B.39. Unscheduled DNA synthesis (UDS) test with mammalian liver cells in vivo [equivalent to OECD TG 486 (OECD 1997c)]	In vivo
	B.49. In vitro mammalian cell micronucleus test [equivalent to OECD TG 487 (OECD 2016o)]	In vitro
	B.58. Transgenic Rodent Somatic and Germ Cell Gene Mutation Assays [equivalent to OECD TG 488 (OECD 2013)]	In vivo
	B.62. In vivo alkaline single-cell gel electrophoresis assay for DNA strand breaks (comet assay) [equivalent to OECD TG 489 (OECD 2016p)]	In vivo
Acute systemic toxicity	B.1 bis. Acute oral toxicity—Fixed dose procedure [equivalent to OECD TG 420 (OECD 2002a)]	In vivo
	B.1 tris. Acute oral toxicity—Acute toxic class method [equivalent to OECD TG 423 (OECD 2002b)]	In vivo
	B.2. Acute toxicity (Inhalation) [equivalent to OECD TG 403 (OECD 2009a)]	In vivo
	B.3. Acute toxicity (Dermal) [equivalent to OECD TG 402 (OECD 2017c)]	In vivo
	B.52. Acute Inhalation Toxicity—Acute Toxic Class Method [equivalent to OECD TG 436 (OECD 2009b)]	In vivo
	OECD TG 425 (OECD 2008b) on Acute oral toxicity: up-and-down procedure)	In vivo
	OECD TG 433 (OECD 2018g) Acute Inhalation Toxicity: Fixed Concentration Procedure	In vivo
Skin sensitisation	B.6: in vivo Guinea Pig test method [equivalent to OECD TG 406 (OECD 1992), comprising the Guinea Pig Maximisation Test (GPMT) and the Buehler Test]	In vivo
	B.42. Local lymph node assay (LLNA) [equivalent to OECD TG 429 (OECD 2010b)]	In vivo
	B.50. Local lymph node assay: DA [equivalent to OECD TG 442A (OECD 2010c)]	In vivo
	B.51. Local lymph node assay: BrdU-ELISA [equivalent to OECD TG 442B (OECD 2018i)]	In vivo
	B.59: Direct peptide reactivity assay (DPRA) addressing the key event on ‘covalent binding to proteins’ of the AOP for skin sensitisation [equivalent to OECD TG 442C (OECD 2020b)]	In vitro
	Amino acid Derivative Reactivity Assay (ADRA) [included in OECD TG 442C (OECD 2020b)]	In vitro
	B.60: ARE-Nrf2 Luciferase Test Method (equivalent to OECD TG 442D) (OECD 2018j)	In vitro
	B.71: In vitro skin sensitisation assays addressing the key event on ‘activation of dendritic cells’ of the AOP for skin sensitisation (equivalent to OECD TG 442E) (OECD 2018k)	In vitro
Repeated dose toxicity	B.7. Repeated dose (28 days) toxicity (Oral) [equivalent to OECD TG 407 (OECD 2008a)]	In vivo
	B.8. Repeated dose (28 days) toxicity (Inhalation) [equivalent to OECD TG 412 (OECD 2018d)]	In vivo
	B.9. Repeated dose (28 days) toxicity (Dermal) [equivalent to OECD TG 410 (OECD 1981a)]	In vivo
	B.26. Sub-chronic oral toxicity test repeated dose 90-day oral toxicity study in rodents [equivalent to OECD TG 408 (OECD 2018c)]	In vivo
	B.27. Sub-chronic oral toxicity test repeated dose 90-day oral toxicity study in non-rodents [equivalent to OECD TG 409 (OECD 1998)]	In vivo
	B.28. Sub-chronic dermal toxicity study 90-day repeated dermal dose study using rodent species [equivalent to OECD TG 411 (OECD 1981b)]	In vivo
	B.29. Sub-chronic inhalation toxicity study 90-day repeated inhalation dose study using rodent species [equivalent to OECD TG 413 (OECD 2018e)]	In vivo
	B.30. Chronic Toxicity test [equivalent to OECD TG 452 (OECD 2018n)]	In vivo
	B.33. Combined Chronic Toxicity/Carcinogenicity Studies [equivalent to OECD TG 453 (OECD 2018o)]	In vivo
	B.38. Delayed neurotoxicity of organophosphorus substances 28 day repeated dose study [equivalent to OECD TG 419 (OECD 1995)]	In vivo
	B.43. Neurotoxicity study in rodents [equivalent to OECD TG 424 (OECD 1997a)]	In vivo
	Combined repeated dose toxicity study with the reproduction/developmental toxicity screening test (OECD TG 422) (OECD 2016f)	In vivo
Carcinogenicity	B.32. Carcinogenicity test [equivalent to OECD TG 451 (OECD 2018m)]	In vivo
	B.33. Combined chronic toxicity/Carcinogenicity test [equivalent to OECD TG 453 (OECD 2018o)]	In vivo
	B.21. In vitro Mammalian cell transformation test	In vitro
Reproductive/ developmental toxicity	B.31. Prenatal developmental toxicity Study [equivalent to OECD TG 414 (OECD 2018f)]	In vivo
	B.35. Two-Generation Reproduction toxicity Study [equivalent to OECD TG 416 (OECD 2001)]	In vivo
	B.53. Developmental Neurotoxicity study [equivalent to OECD TG 426 (OECD 2007a)]	In vivo
	B.54. Uterotrophic Bioassay in rodents [equivalent to OECD TG 440 (OECD 2007b)]	In vivo
	B.55. Hershberger bioassay in rats [equivalent to OECD TG 441 (OECD 2009c)]	In vivo
	B.56. EOGRTS [equivalent to OECD TG 443 (OECD 2018l)]	In vivo
	OECD TG 421 (Reproduction/Developmental Toxicity Screening Test) (OECD 2016e)	In vivo
	OECD TG 422 (Combined Repeated Dose Toxicity Study with the Reproduction/Developmental Toxicity Screening Test) (OECD 2016f)	In vivo
ADME/TK	B.36. Toxicokinetics [equivalent to OECD TG 417 (OECD 2010a)]	In vivo
	B.44. Skin absorption: In vivo method [equivalent to OECD TG 427 (OECD 2004a)]	In vivo
	B.45. Skin absorption: In vitro method [equivalent to OECD TG 428 (OECD 2004b)]	In vitro

For cosmetic ingredients, skin corrosion/skin irritation and serious eye damage/eye irritation should be assessed using the adopted in vitro methods already specified in Regulation 440/2008 (2019b) (Table 2), together with in chemico/in silico [i.e., (Q)SAR]. Data obtained from the Draize rabbit test (EC B.4, OECD TG 404) should be provided when available if the test was performed before the animal testing ban, or if the data were obtained to be in compliance with other legislations (e.g., REACH). In SCCS/1602/18 (2018) it is further commented that currently available replacement alternatives for serious eye damage/irritation testing cannot identify any mild eye irritancy potential. Additionally, for eye irritation, no validated alternative method fully replacing the in vivo test (OECD TG 405, EC B.5) can be identified. Therefore, two separate decision trees for eye irritation were put forward: (i) a decision tree specific for hazard identification of the neat cosmetic ingredient (to classify irritant vs non-irritant, using physicochemical properties, read-across data, (Q)SAR results and in vitro eye irritation data); (ii) a decision tree for risk assessment of the neat ingredient in its final formulation(s) (i.e., formulation’s eye irritancy measured in one or more in vitro eye irritation test(s) vs measured irritancy of a benchmark control, including a confirmatory formulation test with human volunteers).

### Photo-induced toxicity

CLP (2020f) and REACH (2020g) do not specifically ask for photo-toxicity testing and/or labelling requirements. In the most recent SCCS Notes of Guidance (NoG), one in vitro test method, listed in Regulation 440/2008 (2019b) as test method B.41 In vitro 3T3 NRU Phototoxicity Test [equivalent to OECD TG 432 (OECD 2004c)] is indicated as a mandatory in vitro method to assess photo-induced toxicity, when in the exposure assessment (3.3 in NoG) under “functions and uses of cosmetic ingredients” (3.3.1 in NoG) of the dossier submitted, it is shown that exposure to sunlight is possible and the chemical structure indicates the possibility of UV absorption (aromatic groups, double bounds, etc.) and a UV spectrum shows UV absorption. If the UV spectrum does not show UV absorption, there cannot be photo-induced toxicity. As waving principles photo-toxicity tests should not be performed if the test material absorbs at wavelengths < 313 nm, and absorption at longer wavelengths is insufficient. For all UV-filters, (in Annex VI of Reg 1223/2009) the 3T3 NRU Phototoxicity test, comparing the cytotoxicity of a chemical tested in the presence and in the absence of exposure at a non-cytotoxic dose of ultraviolet/visible (UV/VIS) light (SCCS 2018), is mandatory (section 3.2.6 in NoG).

Apart from the 3T3 NRU PT [EC B.41, OECD TG 432 (OECD 2004c)], a reconstructed human skin model can be used as a second tier in particular in case of false positives in the 3T3 NRU PT to evaluate effects (checking for the solvents used), and the use of in chemico/in silico [i.e., (Q)SAR] is encouraged (SCCS 2018). While, to date, validated in vitro methods for the detection of photo-sensitisation are not yet available, chemicals showing photo-allergic properties are likely to give positive reactions in the 3T3 NRU PT test.

At present, no official guideline-based protocols for photo-irritation and photo-sensitisation testing in vivo have been evaluated (SCCS 2018).

To assess photo-mutagenicity/photo-clastogenicity, several assays have been adapted to a combined treatment of chemicals with UV–Visible light (EC 2003), including: (1) bacterial and yeast mutation assays, (2) tests for detecting clastogenicity, (3) tests for detecting gene mutations in mammalian cells, and (4) tests for detecting aneugenicity in mammalian cells in vitro. Other available tests are: the photo-Ames test, the photo HPRT/photo-mouse lymphoma assay, the photo-micronucleus test, the photo-chromosome aberration test and the photo-Comet assay (all to be evaluated on a case-by-case basis) (Brendler-Schwaab et al. 2004). In chemico/in silico methods are also indicated.

There is no requirement for photo-mutagenicity testing when the phototoxicity tests are negative, or if the compounds have a Molar Extinction Coefficient (MEC) below 1000 L mol^−1^ cm^−1^ (EFSA 2020).

### Mutagenicity/genotoxicity

According to CLP Regulation (2020f), hazard categories for germ cell mutagens are related to substances that may cause mutations in the germ cells of humans that can be transmitted to the progeny. Since human data are not available, the results obtained with mutagenicity or genotoxicity tests in vitro and in mammalian somatic and germ cells in vivo are used in classifying substances and mixtures within this hazard class. Category 1 (accounting for subcategories 1A and 1B) identifies substances known to induce heritable mutations (Cat 1A) or to be regarded as if they induce heritable mutations in the germ cells of humans (Cat 1B). Category 2 applies to substances that may induce heritable mutations in the germ cells, therefore causing concern for humans.

For a comprehensive coverage of the potential mutagenicity of a substance, information on gene mutations (base substitutions and deletions/additions), structural chromosome aberrations (breaks and rearrangements, defined as clastogenicity) and numerical chromosome aberrations (loss or gain of chromosomes, defined as aneuploidy) is required (EC 1223/2009) (EC 2020e; ECHA 2017b).

Under REACH (2020g), the assessment of mutagenicity follows a stepwise approach, which starts with a battery of in vitro tests, followed up by appropriate in vivo testing in case one or more of the in vitro tests are positive. The in vitro studies for mutagenicity include an in vitro gene mutation study in bacteria (Ames test), an in vitro cytogenicity study in mammalian cells (i.e., an in vitro chromosome aberration study or an in vitro micronucleus study) and, if both in vitro tests are negative, an in vitro gene mutation study in mammalian cells should be performed. If there is a positive result in any of the above in vitro studies and there are no results available from an appropriate in vivo study already, an appropriate follow-up in vivo study in somatic cells must be proposed by the registrant. In some cases, a second in vivo somatic cell test may be necessary depending on the quality and relevance of all available data. If there is a positive result from an in vivo somatic cell study, the potential for germ cell mutagenicity should be considered on the basis of all available data, including TK information (if available). Moreover, as for any other endpoint under REACH, the information required for a substance depends on its volume (tpy) of production or importation.

Several in vitro and in vivo test methods and OECD TGs for mutagenicity and genotoxicity are indicated in Regulation (EC) No 440/2008 (2019b), as summarised in Table 2.

To assess the potential for mutagenicity of a cosmetic substance (EC 1223/2009) (EC 2020e), two tests in particular are recommended: the Bacterial Reverse Mutation Test, Ames (OECD TG 471) (OECD 1997b), to assess gene mutations, and the In vitro Micronucleus Test (OECD TG 487) (OECD 2016o), to assess both clastogenicity and aneugenicity.

In cases where the bacterial reverse mutation test is not suited, as in the case of nanoparticles, a revised genotoxicity test battery, which includes in vitro mammalian cell mutagenicity and clastogenicity assessments, has been recommended (Elespuru et al. 2018).

If the results from both tests are clearly negative in adequately performed tests, it is very likely that the substance has no mutagenic potential. Likewise, if the results from both tests are clearly positive, it is very likely that the substance has mutagenic potential. In both cases, further testing is not necessary. If one of both tests is positive, the substance is considered an in vitro mutagen, and further in vitro testing is needed to exclude the potential mutagenicity of the substance under investigation. A toolbox for the evaluation in a Weight-of-Evidence (WoE) approach has been proposed in the SCCS/1602/18 (2018), which includes among others: the comet assay in mammalian cells, comet or micronucleus assay on 3D-reconstructed human skin, the Hen’s Egg test for Micronucleus Induction (HET-MN), mechanistic investigations (e.g., toxicogenomics) or internal exposure (TK), Reporter gene assays based on human, animal or bacterial cells (Pfuhler et al. 2020). For chemicals that are primarily associated with dermal exposure, the use of reconstructed human skin models has been explored and protocols have been developed for a reconstructed skin micronucleus test (RSMN) (Curren et al. 2006; Mun et al. 2009) and a RS Comet assay (i.e., 3D Skin Comet) (Reisinger et al. 2018) based on the best suited skin tissues (Curren et al. 2006; Pfuhler et al. 2011; Reisinger et al. 2018). The development of OECD test guidelines based on these tests is currently ongoing.

### Acute systemic toxicity

In the Regulation (EC) No 1272/2008 (CLP) (2020f), acute toxicity hazard categories and acute toxicity estimates defining the respective categories are based on animal data, while categories for specific target organ toxicity after single exposure are based on evidence from humans and/or from experimental animals. Animal studies to assess adverse effects and LD_50_ or LC_50_ value of tested compounds (which may result from a single exposure, usually carried out with high doses of the test substance), are thought to allow determination or estimation of a range of severe acute toxic effects including mortality. Substances can be allocated to one of four toxicity categories based on acute toxicity by the oral, dermal or inhalation route according to the numeric criteria.

Under REACH (2020g), and as described in the ECHA Guidance (2017b), the assessment of acute systemic toxicity is among the standard information requirements for substances manufactured or imported into the EU in quantities of 1 tonne or more per year (tpy), and standard information requirements are specified in Annexes VII and VIII. Acute toxicity testing is not required if the substance is corrosive to the skin. In particular, as indicated under Annex VII (≥ 1 tpy), acute toxicity study(ies) via the oral route of exposure is(are) required, and waiving is allowed if a study on acute toxicity by the inhalation route is available. For substances manufactured or imported into the EU in quantities of ≥ 10 tpy (under Annex VIII), in addition to acute toxicity study(ies) via the oral route of exposure, information on at least one other route of exposure is requested, depending on the nature of the substance and the likely route of human exposure. As described in Column 2 of section 8.5.3 of Annex VIII, waiving of acute dermal toxicity testing is further allowed if: (i) the substance does not meet the criteria for classification for acute toxicity or STOT-SE (specific target organ toxicity-single exposure) by the oral route, and (ii) no systemic effects have been observed in in vivo studies with dermal exposure (e.g., skin irritation, skin sensitisation) or, in the absence of an in vivo study by the oral route, no systemic effects after dermal exposure are predicted on the basis of non-testing approaches [e.g., read across, (Q)SAR studies]. In line with this, WoE-based adaptation to the standard information requirement may be adopted for acute oral toxicity studies, particularly for substances to be registered at Annex VIII tonnage level and above (i.e., registrations at > 10 tpy), for which an oral sub-acute toxicity study (OECD TG 407) (OECD 2008a) or the combined repeated dose toxicity study with the reproduction/developmental toxicity screening test (OECD TG 422) (OECD 2016f) is required. This WoE adaptation proposed by ECHA (ECHA 2017b) applies to low toxicity substances (i.e., those that are not to be classified for acute oral toxicity). Further considerations regarding these adaptation rules are also discussed in Buesen et al. 2018; Gissi et al. 2017, 2018; Graepel et al. 2016.

According to the ECHA Guidance (2017b), derivation of LD_50_ or LC_50_ values is no longer considered essential. Indeed, some of the current standard acute systemic toxicity TGs [e.g., EU B.1 bis/OECD TG 420 (OECD 2002a) and OECD TG 433 (OECD 2018g)], use signs of non-lethal toxicity (rather than mortality). These test methods should be preferred as they present advantages over the other guidelines in terms of animal welfare.

Recommended test methods, as indicated in Regulation (EC) No 440/2008 (2019b), and corresponding OECD TGs for acute systemic toxicity are summarised in Table 2.

As per Regulation (EC) No 1223/2009 (Cosmetic Products Regulation) (2020e), acute systemic toxicity plays in practice a limited role for the cosmetics industry. Ingredients used in this sector essentially do not raise the risk of acute systemic toxicity and sufficient information is often available from repeated dose studies if conducted before 2013. Additionally, any possible impacts on the toxicological profile due to particle sizes, including nanomaterials, impurities of the substances and raw material used, and interaction of substances should be considered, and validated alternative non-animal methods applied.

According to the Notes of Guidance SCCS/1602/18 (2018), validated (animal-free) replacement methods for acute systemic toxicity are not available. However, data on acute systemic toxicity are not mandatory for assessing the safety of cosmetic ingredients for consumer uses. A WoE approach [e.g., data from chemical grouping/read-across, (Q)SAR, in vitro studies, or repeated dose toxicity studies] may be sufficient to drive conclusions on the safety of cosmetic products for acute systemic toxicity.

As already mentioned under  "Skin corrosion and irritation and serious eye damage/eye irritation" section, OECD GD 237 opens the possibility to waive animal studies where the results of validated in vitro tests or alternative approaches are adequate to draw a conclusion regarding the classification of an acute hazard for a test chemical. These waiving principles are applicable to mammalian acute toxicity (oral, dermal and inhalation route), eye and skin irritation and skin sensitisation, and although they were mainly intended for pesticides, they can be extended to other chemicals, formulations and biological materials. The approaches outlined in OECD GD 237 should be used by regulatory jurisdictions as part of the WoE to determine the need for a mammalian acute toxicity study and establish appropriate classification and/or labelling.

### Skin sensitisation

Assessment of categories and subcategories for skin sensitisers under CLP (2020f) is done considering evidence derived from effects seen in humans and/or animal tests. Skin sensitisers are classified as Category 1. If data allow, optional subcategorisation of sensitisers into subcategories 1A (strong sensitisers) and 1B (other skin sensitisers) can be performed. As a general comment, when considered in the context of a WoE approach, evidence from animal studies is usually more reliable than evidence from human exposure, since the latter is usually derived under less controlled studies. Human evidence may derive from clinical experience, diagnostic patch testing, and other tests designed to confirm the absence of sensitisation potential under expected exposure conditions. Human tests for the purpose of hazard identification are not conducted in the EU because considered unethical.

REACH information requirements for skin sensitisation have been recently revised [Section 8.3 of Annex VII, as of May 2017 (EC 2017a)] and this information should come from: (i) in vitro/in chemico data addressing the three key events (KEs) described in the skin sensitisation Adverse Outcome Pathway (AOP) (i.e., molecular interaction with skin proteins, inflammatory response in keratinocytes, activation of dendritic cells) (Landesmann and Dumont 2012; OECD 2012); and (ii) an in vivo study, normally a Local Lymph Node Assay (LLNA) [described in OECD TG 429 (OECD 2010b)], in case the in vitro/in chemico studies are not applicable for the substance, or are not adequate for classification and risk assessment. In case a substance is considered a skin sensitiser, the revised REACH requirements also introduce the need to assess whether it can be presumed to have the potential to produce significant sensitisation in humans (i.e., GHS /CLP Cat. 1A).

The ECHA guidance document (ECHA 2017b) for this endpoint has been revised to inform about the recent adoption or revision of several EU test methods and/or OECD TGs for skin sensitisation. Additionally, information about the use of non-testing data has been updated to reflect ECHA’s current approach to dossier evaluation. The testing and assessment strategy for skin sensitisation has also been updated, and now it foresees the use of non-animal test methods addressing AOP KEs for generating adequate information. According to Annex VI, the registrant should gather and evaluate all existing available information before considering further testing. This includes structural considerations, physico-chemical properties, (Q)SAR, information from structurally similar substances, in vitro/in chemico data, animal studies, and human data. For classified substances, information on exposure, use and risk management measures should also be collected and evaluated to ensure that potential risks are identified and adequate risk management measures are taken.

The in vivo and in vitro test methods (and OECD TGs) for skin sensitisation (Regulation 440/2008 (2019b)) are summarised in Table 2. In particular, B.71: In vitro skin sensitisation assays (equivalent to OECD TG 442E) addresses the activation of dendritic cells, one KE in the AOP for skin sensitisation (Landesmann and Dumont 2012; OECD 2012), and provides three in vitro test methods addressing mechanisms under the same KE: (i) the human Cell Line Activation Test (or h-CLAT method), (ii) the U937 Cell Line Activation Test (or U-SENS), and (iii) the Interleukin-8 Reporter Gene Assay (or IL-8 Luc assay).

For testing of cosmetics ingredients, skin sensitisation is considered among the most relevant endpoints due to the high frequency of allergic reactions among the undesirable effects of cosmetic products. Notably, recent efforts have been made by the cosmetic industry to develop a non-animal, next generation risk assessment (NGRA) framework for the assessment of skin sensitisers (Gilmour et al. 2020).

### Repeated dose toxicity

According to the CLP Regulation (2020f), categories for specific target organ-toxicity—repeated exposure are based on evidence from humans (although rarely available) and/or from in vivo laboratory animal studies. Under REACH, the standard information requirements for repeated dose toxicity are in vivo studies (in rats) of increasing minimum duration as the tonnage band increases. The oral route is the most common, but substance properties and the relevant exposure route for humans need to be taken into account. The standard information requirements on repeated dose toxicity are specified in REACH Annexes VIII-X. Information on a sub-acute (28-day) study is needed at Annex VIII (10–100 tpy) level. At the next tonnage band, a longer study, i.e., sub-chronic (90-day) study, is required. In addition, further studies may be needed at Annex levels IX and X to address concerns related to longer exposure duration, different route of administration and/or specific toxicological investigations, such as immunotoxicity or neurotoxicity. Long-term chronic toxicity studies may be needed based on human exposure considerations. In the context of REACH, the benchmark dose [BMD, defined as the dose corresponding to a *‘specific change in an adverse response compared to the response in unexposed subjects*’ (Dakeishi et al. 2006)] may also be used, and species-specific information, e.g., on respiration rates and body weight, enable extrapolation between studies with different exposure routes.

Importantly, the ECHA Guidance (2017b) describes the use of an Integrated Testing Strategy (ITS) for repeated dose toxicity. In particular, testing for repeated dose toxicity is not required for chemicals produced at tonnage levels less than 10 tpy, whilst at higher production volumes, standard data requirements are increased with each tonnage.

As indicated in Regulation (EC) No 440/2008 (2019b), current standard test methods and corresponding OECD TGs are all in vivo studies (Table 2).

As outlined in both Regulation (EC) No 1223/2009 (Cosmetic Products Regulation) (2020e) and SCCS/1602/18 (2018), evaluation of systemic toxicity is a key element for cosmetic ingredients, which are repeatedly in contact with human skin and mucosa. If studies of only 28-day duration are available, a default assessment factor of three to extrapolate from subacute (28 days) to subchronic (90 days) toxicity may be used in the calculation of the Margin of Safety (MoS), as also applied under REACH (ECHA 2012). The inhalation route is only rarely used in repeated dose toxicity testing of cosmetic ingredients, unless a cosmetic product is intended to be used in an aerosolised, sprayable, or powdered form. If the dose regimen of a study was 5-day treatment per week, the derived dose-descriptor corrected by a factor of 5/7 is normally used. SCCS recognises that the BMD can be used as an alternative to the No Observed Adverse Effect Level (NOAEL) approach for deriving a Point of Departure (PoD), which is defined as the point on a toxicological dose–response curve corresponding to an estimated low effect level or no effect level (ChemSafetyPro 2018). The 28-day and 90-day oral toxicity tests in rodents are the most commonly used repeated dose toxicity tests. Preferably, studies of 90 days or more should be used in safety assessments. In a number of cases, dermal repeated dose toxicity studies are present among the submitted data for the cosmetic ingredients listed in Annexes III-VI of Cosmetic Products Regulation, as for example in the case of UV-filters.

### Carcinogenicity

Under CLP (2020f), hazard categories for carcinogens are largely based on human (if available) and/or animal evidence. Category 1 accounts for known or presumed human carcinogens on the basis of epidemiological and/or animal data. A substance may be further distinguished as category 1A (i.e., carcinogenic potential for humans, based on human evidence), or category 1B (i.e., presumed carcinogenic potential for humans, based on animal evidence). Category 2 is assigned to suspected human carcinogens, and this classification is done on the basis of evidence obtained from human and/or animal studies, which is not convincing enough to place the substance in Category 1A or 1B.

REACH (2020g) requires a carcinogenicity test for substances falling under Annex X (≥ 1000 tpy), in case: (i) of widespread dispersive use, or when there is evidence of frequent or long-term human exposure, and (ii) if the substance is classified for mutagenicity (germ cell mutagen category 3 under CLP, now category 2), or there is evidence from the repeated dose study(ies) that the substance is able to induce hyperplasia and/or pre-neoplastic lesions.

If the substance is classified as mutagen category 1A and 1B, the default presumption would be that a genotoxic mechanism for carcinogenicity is likely. In these cases, a carcinogenicity test will normally not be required, according to the standard information requirement (Annex X).

Proposals for conducting a carcinogenicity test should be made with regard to the potential risk to human health and with consideration of the actual or intended production and/or use pattern. However, REACH also requires that carcinogenic substances at all tonnage levels be identified as substances of high concern, taking into account information from all available relevant sources (non-human and human, non-testing and testing data), which can inform on hazard identification, underlying modes of action or carcinogenic potency. In addition, the classification and labelling as listed in Annex VI of CLP Regulation is legally binding and can trigger further assessment under REACH to decide if the substance should be formally identified as a substance of very high concern (SVHC) (Madia et al. 2016).

The ECHA Guidance (2017b) proposes a testing strategy entailing the following three steps for the assessment of carcinogenicity for substances at each of the tonnage levels specified in Annexes VII to X of REACH: (i) gather and assess all available test and non-test data from read-across and/or proper chemical category (chemical grouping) and suitable predictive models, and examine the WoE that relates to carcinogenicity; (ii) consider whether the standard information requirements are met; (iii) ensure that the information requirements of Annexes VII and VIII are met, and make proposals to conform to Annexes IX and X (whether further tests are needed to fulfil requirements under Annexes IX and X).

In case a carcinogenicity study needs to be conducted, a testing proposal needs to be submitted to the agency as specified in REACH. For substances at annex X, predictive techniques, such as chemical grouping and read-across, and the use of (Q)SARs may be supplemented with in vitro or alternative shorter-term in vivo studies to circumvent the need for a carcinogenicity study (ECHA 2017b).

Different sources of information may enable drawing inferences regarding the potential of a chemical to be carcinogenic to humans. In particular, non-human data, including non-testing data, testing data (both in vitro and animal), human data, and information on exposure, use and risk management should be considered (paragraph R.7.7.10, Information sources on carcinogenicity) (ECHA 2017b).

In the Regulation (EC) No 440/2008 (2019b), two in vivo tests are described: B.32. Carcinogenicity test [equivalent to OECD TG 451 (OECD 2018m)], and B.33. Combined chronic toxicity/Carcinogenicity test [equivalent to OECD TG 453 (OECD 2018o)], and one in vitro test: the B.21. In vitro Mammalian cell transformation test (see Table 2). At present, no validated (animal-free) replacement methods included in OECD TGs to study carcinogenicity are available.

As for industrial chemicals under REACH, also for cosmetics ingredients, genotoxicity information is the main driver for consideration of carcinogenicity.

Two OECD Guidance Documents (GDs) on in vitro Cell Transformation Assays (CTA) have been adopted: CTA in Syrian Hamster Embryo (SHE) cells performed at pH 6.7 and at pH 7.0 (OECD GD 214) (OECD 2015b), and CTA in Bhas 42 cell line (OECD GD 231) (OECD 2016b).

As suggested in the SCCS Notes of Guidance (SCCS 2018), a positive result in one of the in vitro genotoxicity tests may be indicative to consider a substance as a putative carcinogen. This indication may be further supported by a positive result in cell transformation assays. However so far, there are no specific requirements to obtain information on non-genotoxic carcinogenicity as such, and many non-genotoxic carcinogens may remain unidentified (Jacobs et al. 2016). According to the SCCS Notes of Guidance (SCCS 2018), also in vitro toxicogenomics can be used in a WoE approach, especially for the detection of non-genotoxic carcinogens. CTA in combination with other existing information and toxicogenomics approaches may be considered as part of integrated approaches to testing and assessment (IATA). Further information on the status of in vitro carcinogenicity testing can be found in (Adler et al. 2011; Jacobs et al. 2020; Madia et al. 2014, 2016; Worth et al. 2014).

### Reproductive and developmental toxicity

CLP criteria for hazard categories for reproductive toxicants are either based on evidence from humans (rarely available) and/or data from animal studies (2020f). Category 1A is assigned to known human reproductive toxicants based on evidence in humans, and category 1B is assigned to chemicals that are presumed human reproductive toxicants based on data from animal studies. When there is mechanistic information that raises doubt about the relevance of the effects for humans, classification in Category 2, which identifies suspected human reproductive toxicants, may be considered more appropriate. Moreover, classification as a reproductive toxicant is made on the basis of a WoE assessment, i.e., all available information is considered together. This information may be derived from epidemiological studies and case reports in humans and specific reproduction studies in animals that investigate fertility, sexual function and developmental effects in offspring along with sub-chronic, chronic and special studies in animals that provide relevant information regarding toxicity to reproductive and related endocrine organs.

Under REACH (2020g), the reproductive toxicity of a substance is primarily assessed by means of three different studies: (i) a reproduction/developmental toxicity screening test (e.g., OECD TG 421/422), (ii) prenatal developmental toxicity studies in two species, and (iii) an extended one-generation reproductive toxicity study (EOGRTS). It should be considered that at Annex VII, none of these tests need to be provided, while at Annex VIII, a screening study is required as a minimum, with the proposal to consider performing a prenatal developmental toxicity study if there are any indications of concern for this endpoint from existing information. The EOGRTS would normally only be required at Annex X but could be triggered at lower tonnages (Annexes VIII or IX) on the basis of concerns of potential adverse effects from existing information. Theoretically, in exceptional cases, information from an EOGRTS in a second species or strain may be legally required at Annex X.

The EOGRTS [EC B.56, OECD TG 443 (OECD 2018l)] is now considered the information requirement for reproductive toxicity instead of the two-generation reproductive toxicity study [EC B.35, OECD TG 416 (OECD 2001)] based on an amendment from 2015 (Commission Regulation (EU) 2015/282) (EC 2015a). Although a two-generation reproductive toxicity study is accepted to cover the standard information requirement, instead of an EOGRTS, if initiated before March 13, 2015. EOGRTS offers a number of advantages in comparison to the two-generation reproductive toxicity study, as it assesses a greater number of animals of the first filial generation (F1) and addresses additional parameters, improving the sensitivity and level of information that can be obtained from the test, and may allow a reduction of the number of animals to be used (depending on the study design). The standard information requirement in Annexes IX and X should be limited to the basic configuration of EOGRTS (without extension to include an F2 generation).

Nevertheless, in certain specific cases, where justified, the registrant should be able to propose and ECHA should be able to request the performance of the F2 generation (e.g., on the basis of concerns for endocrine disruption), as well as the developmental neurotoxicity (DNT) and developmental immunotoxicity (DIT) cohorts. DNT and DIT are regarded as important and relevant developmental toxicity endpoints, which could be further investigated. However, analysing the DNT and DIT cohorts entails significant additional costs as well as subjecting animals to additional experiments. Currently, analysis of DIT and/or DNT cohorts is only requested subject to specific concern-driven triggers (see “Developmental neurotoxicity (DNT)” and “Immunotoxicity and developmental immunotoxicity (DIT)” sections).

In REACH, studies on reproductive and developmental toxicity are required from Annex VIII through Annex X, and the standard information requirements are cumulative (i.e., requirements at higher tonnage levels add to the information requirements at lower tonnage levels). If a substance is known to have an adverse effect on fertility, meeting the criteria for classification as Repr Cat 1A/1B, and the available data are adequate to support a robust risk assessment, then no further testing for sexual function and fertility will be necessary. However, testing for developmental toxicity must be considered. With regard to substances known to cause developmental toxicity and classified as Repr Cat 1A/1B, no further testing for developmental toxicity will be necessary, although testing for effects on fertility must be considered. In cases where there are serious concerns about the potential for adverse effects related to fertility or development, the registrant may propose an EOGRTS (Annex IX, Section 8.7.3) and/or a pre-natal developmental toxicity study (Annex IX, Section 8.7.2), as appropriate, instead of the screening study to address the concern(s). If there are no adverse effects leading to a concern for development, a pre-natal developmental toxicity study may not be used to fulfil the requirement for a reproductive screening study.

The ECHA Guidance (ECHA 2017b) further comments on the applicability of an ITS for reproductive toxicity, which is defined as an approach that combines one or more non-animal methods with animal studies to fulfil the information requirements, or could include only non-animal methods if they together covered all key aspects of reproductive toxicity. However, the use of non-animal methods should be assessed on a case-by-case manner, ensuring that the obtained results cover all of the key aspects of reproductive toxicity and are suitable for both risk assessment (e.g., derivation of NOAEL) and classification and labelling.

Table 2 summarises the test methods (Regulation 440/2008 (2019b)) and corresponding OECD TGs suitable to assess reproductive and developmental toxicity.

With regard to cosmetic ingredient safety assessment, the one or two-generation reproduction toxicity test (or the EOGRTS) were the most commonly performed in vivo reproductive toxicity studies before the animal testing ban.

Three alternative embryotoxicity-related methods are currently available: (1) the Whole Embryo Culture test (WEC), (2) the MicroMass test (MM), and (3) the Embryonic Stem cell Test (EST), which can all be used to identify strong embryotoxic substances (Balls and Hellsten 2002; Spielmann et al. 2006). At OECD level, a detailed review paper on “Pluripotent stem cell assays: Modalities and applications for predictive developmental toxicity” is currently under development.

Other in vitro methodologies, covering male and female fertility, implantation and pre- and postnatal development have been and are being developed, such as under ReProTect (http://www.reprotect.eu/) or the EURION cluster (https://eurion-cluster.eu/). It should be considered that, to date, validated animal-free methods accepted as a full replacement are not available (Adler et al. 2011; Worth et al. 2014), and that the available alternative methods are not able to mimic all of the various developmental stages, therefore a battery of tests will be needed. However, a more radical change towards next generation risk assessment may allow to move away from prediction of current toxicity classes to prediction of likely safe doses, as indicated in the OECD GD 275 (OECD 2017a).

### Absorption, distribution, metabolism and excretion (ADME) and toxicokinetics (TK)

Information on the biological fate of a chemical in the body plays an important role in human safety assessment. While there are few explicit requirements in EU chemicals legislation for the generation of TK data (i.e., in vitro, in vivo measurements or computational predictions), the use of these data to support the assessment of systemic toxicity is widely recommended in regulatory guidance, although not consistently required in regulations (Bessems et al. 2015). For instance, ADME/TK information is required under the Biocidal Products (Regulation (EU) No 528/2012) (EC 2012) and Plant Protection Products [Regulation (EC) No 1107/2009 (EC 2009) and Commission Regulation (EU) No 283/2013 (EC 2013b)] (which are out of the scope of this document), and the EU Plant Protection regulation also requires the generation of human in vitro biotransformation data to compare with rodent data and studies. However, this is not the case for other regulations as briefly explained.

There are no CLP categories for TK and the CLP Regulation does not specifically require the assessment of ADME and TK (2020f). However, ADME and TK data may be used in a WoE approach to classify, lower the classification or abstain from classification for a particular toxicodynamic (TD) endpoint. For the classification of substances as carcinogens, all available information regarding the physicochemical, TK and TD properties of the substances, as well as information on structure activity relationships, should be taken into account to undertake classification.

Under REACH (2020g), TK studies in vivo are not required; however, all available information should be provided, including TK information. Importantly, human health hazard assessment shall consider ADME and TK of substances. Even though TK is not a toxicological endpoint and is not specifically required by REACH, the generation of TK information can help interpret data, assist testing strategy and study design, as well as category development, thus helping to optimise test designing. Furthermore, under REACH, TK data would be very useful for assessing read-across and categories, but as this is not a standard information requirement, that information is rarely available.

The ECHA Guidance (ECHA 2017b) reports many examples of recommendations on the use of TK data that would replace default assessment factors (e.g., Sections R.7.12 and R.8.4 in Chapters R.7.C and R.8, respectively). The guidance highlights that TK studies may be helpful in the evaluation and interpretation of repeated dose toxicity data (e.g., in relation to accumulation of a substance or its metabolites in certain tissues or organs), as well as in relation to mechanistic aspects of repeated dose toxicity and species differences. TK information can also assist in the selection of the dose levels. A very important observation is that TK and potential TD properties based on available data should be considered before undertaking animal tests. Understanding these properties will enable the design of appropriate protocols for the standard tests to be developed, especially with respect to tissue(s) to be investigated, the route of substance administration and the highest dose to be tested. If there is poor understanding of the systemic availability of a test substance, TK investigations or modelling may be necessary.

The three following test methods (and corresponding OECD TGs) for TK are indicated in Regulation 440/2008 (2019b): B.36. Toxicokinetics (in vivo) [equivalent to OECD TG 417 (OECD 2010a)], B.44. Skin absorption: In vivo method [equivalent to OECD TG 427 (OECD 2004a)], and B.45. Skin absorption: In vitro method [equivalent to OECD TG 428 (OECD 2004b)] (Table 2). These EU test methods and OECD TGs generate data for TK, and currently most of them are based on animal procedures as the traditional approach of obtaining whole-body TK parameters. However, by exploiting modern developments in predictive toxicology, there are increasing opportunities to generate human-relevant whole-body TK information using physiologically based kinetic (PBK) models (Paini et al. 2019).

These mathematical models, which represent the body as a set of interconnected compartments linked by blood flow, would enable not only the generation of TK data, but also the integration of human data generated by in silico and in vitro methods for ADME. The lack of standardisation of such methods hampers their regulatory acceptance and use (Bessems et al. 2015). However, there is an on-going international effort at OECD to promote the regulatory use of PBK models based on in silico and in vitro data and body physiological parameters (Sachana 2019).

In relation to cosmetic ingredients, information on TK parameters (e.g., human systemic and dermal exposure, and biotransformation) is recommended (EC 2020e). In particular, with regard to dermal/percutaneous absorption and in specific cases, data from in vivo studies that have been carried out before the animal testing ban, or data from in vitro biotransformation studies are required (SCCS 2018), to prove or to exclude certain adverse effects (e.g., EC B.44, 45; OECD TG 427, TG 428). For dermal absorption, it should be considered whether the formulation can affect compound bioavailability.

With regard to in vitro dermal absorption of cosmetic ingredients, some basic criteria have been provided when performing in vitro dermal absorption studies, along with rules to follow in case no dermal absorption studies are available (e.g., regarding the amounts to be applied and what to do in case the basic criteria have not been followed) (SCCS 2010).

For substances with very low dermal absorption and limited permeation (such as colourants or UV-filters with high molecular weight and low solubility), the epidermis may be excluded as a route of entry (WHO 2006). For nanomaterials, it should be ascertained whether the substance absorbed through the skin is in nanoparticle form or in a dissolved chemical state.

Besides the determination of TK parameters of the parent chemical, it is also essential to obtain accurate profiles of metabolites that could be more potent than the parent compound. Cells and cell fractions or organ specimens from human sources, although limited, are available, together with 3D cultures to preserve metabolic capacity and regulation of xenobiotic metabolising enzymes. Additionally, the use of*-*to-in vivo extrapolation (IVIVE) and PBK modelling is encouraged to translate external exposures into an internal (target) dose in the body and vice versa (Yoon et al. 2012). PBK models are increasingly being used to aid: (i) extrapolation within and between species (variability issues), (ii) route-to-route, (iii) dose extrapolation, and (iv) replacement of default assessment factors by more specific, substance-derived factors.

## Toxicity effects for which there are currently no direct information requirements

Apart from the major endpoints described above, current EU regulations do not specifically address more physiologically complex toxicity effects, such as DNT, immunotoxicity and DIT, and endocrine disruption. For instance, according to REACH, neurotoxicity and immunotoxicity studies are only required when concern-driven scientific triggers are observed. On the other hand, with regard to cosmetic ingredients, there are no requirements for the assessment of these effects, or, such effects could be assessed using in vitro tests when needed.

### Developmental neurotoxicity (DNT)

In light of the increasing prevalence of cognitive defects in children [e.g., about 1 in 59 children has been identified with some form of autism (CDC 2018)], it is of pivotal importance to develop better testing strategies to evaluate chemicals for their potential to cause DNT. Current strategies to screen chemicals for their potential to induce DNT are based on animal testing, since there are no regulatory accepted non-animal methods for this purpose. Moreover, testing of DNT for regulatory purposes is not a standard requirement within the EU, and DNT testing [OECD TG 426 (OECD 2007a)] is only performed when triggered based on structure activity relationships or evidence of neurotoxicity in systemic adult studies, such as those associated with repeated dose toxicity and reproductive and developmental toxicity (e.g., 28- and 90-day repeated dose toxicity studies, or the EOGRTS). However, there are intrinsic limitations in this approach. For instance, DNT studies are not often performed upon triggers, and this is often due to their time and overall cost (Rovida and Hartung 2009; Tsuji and Crofton 2012). Additionally, triggers of DNT studies may not represent reliable indicators of DNT, as repeated dose toxicity and reproductive and developmental toxicity studies are conducted in adult animals. In fact, the OECD TG 426 has been used to assess the effects of a limited number of pesticides and industrial chemicals (about 120) (Crofton et al. 2012; Kadereit et al. 2012; van Thriel et al. 2012). For these reasons, only a very limited amount of chemicals has been screened and identified as developmental neurotoxicants (Bjorling-Poulsen et al. 2008; Grandjean and Landrigan 2006; Smirnova et al. 2014), and alternative methodologies suitable to more rapidly and cost-effectively screen large numbers of chemicals for their potential to cause DNT in humans are dearly needed (Bal-Price et al. 2018).

It is currently considered that a battery of alternative in vitro methods suitable to capture several key neurodevelopmental processes, combined with in silico approaches [(Q)SAR, read-across, computational modelling] and non-mammalian animal models (e.g., zebrafish, medaka or C. elegans) may pave the way to a more efficient DNT testing (Bal-Price and Fritsche 2018). Under the umbrella of the OECD, an international partnership (EFSA, US EPA, academia, etc.) is currently developing a strategy to enhance regulatory DNT testing using a battery of in vitro assays mainly applied to human neuronal/glial models derived from induced pluripotent stem cells. These in vitro assays are anchored to critical neurodevelopmental processes and KEs identified in DNT AOPs, to gather mechanistic understanding for the development of an IATA. These activities will support the development of an OECD guidance document on the use of alternative methods for DNT testing, including guidance on data interpretation (Sachana et al. 2019).

### Immunotoxicity and developmental immunotoxicity (DIT)

As for DNT, specific information about immunotoxicity and DIT outside the information provided by the general systemic in vivo test methods is not normally required for industrial chemicals or cosmetic ingredients, and the triggers of further testing are considered on a case-by-case basis. Repeated dose toxicity and reproductive and developmental toxicity studies should be performed in a way that allows evaluation of immunotoxicity and/or DIT (e.g., an EOGRTS may be conducted including the immunotoxicity cohort). More specifically, in OECD TG 443 (EOGRTS) (OECD 2018l) it is also specified that ‘*decisions on whether to assess the second generation and to omit the (DNT) cohort and/or (DIT) cohort should reflect existing knowledge for the chemical being evaluated, as well as the needs of various regulatory authoritie*s’, indicating that DIT and/or DNT cohorts should be considered on a case by case basis as part of this TG, also in an effort to maximize information and reduce the number of used animals.

With regard to DIT, early-life environmental insults, by affecting the developing immune system, may significantly impact health of the exposed offspring and, possibly, future generations. Therefore, DIT may play an important role in the onset of non-communicable diseases, as commented by Dietert and co-authors (Dietert 2009; Dietert et al. 2010). DIT has been traditionally assessed in vivo, and most literature reviews on this endpoint have focused on animal research and specific categories of risk factors (e.g., heavy metals). Systematic reviews (and meta-analyses) of human epidemiological studies [such as (Dietert 2014)] are needed to support DIT risk identification. Furthermore, experience gathered across chemical and pharmaceutical industries globally suggests that triggered-based testing approaches together with standard toxicity studies may help evaluate DIT potential (Boverhof et al. 2014). Possible triggers may be: (i) signs of immunotoxicity observed in standard toxicity studies, (ii) a test compound with potential to affect immune functions, (iii) the intended patient population resulting already immunocompromised, (iv) a test compound that is structurally similar to other known immunotoxicants, (v) a drug retained at high concentrations in immune system cells, and (vi) signs of potential immunotoxicity that have been observed in clinical findings (Boverhof et al. 2014).

### Endocrine disruptors (EDs)

Since the late 1990s, endocrine disruptors (EDs) are in the focus of the OECD, with the creation of the advisory group on endocrine disruptors testing and assessment (EDTA AG) and the development of several test methods investigating endocrine activity or ED-related effects. Also the European Commission adopted a Community Strategy for endocrine disruptors in 1999 (EC 1999), which was recently revised (EC 2018c).

According to the 2002 IPCS/WHO broadly accepted definition of EDs, an ED is ‘*an exogenous substance or mixture that alters function(s) of the endocrine system and consequently causes adverse health effects in an intact organism, or its progeny, or (sub)populations*’ (IPSC and WHO 2002).

The main challenge for ED testing is to design test methods complex enough to cover the entire signalling network and the relevant modes of action (MoA). Additionally, current in vivo and non-animal approaches do not easily allow the prediction of effects later in life as a consequence of early life or developmental exposure. Human epidemiological data may be available eventually once health problems have been associated with chemical exposures; however, causal links to specific chemical exposures may be difficult to identify, especially considering the delay in appearance of the health effects in relation to the timing of exposure.

Under REACH (2020g), at the moment, specific information on ED properties is not required; however, reproductive toxicity [e.g., EOGRTS (OECD 2018l)] and organ-related toxicity studies might provide relevant information on ED properties. Additional specific studies during chemical evaluation can be required where concerns about possible ED-related effects are raised. The cosmetics regulation also does not require specific information on ED properties, although a list of potential EDs has been made and the dossiers of these compounds, compiled by the cosmetics industry, are currently under consideration by the SCCS.

The OECD Conceptual Framework for the testing and assessment of EDs has focused on interference with the action and production of sex steroid hormones (oestrogen and androgens) as well as interference with the thyroid hormone system. Some in vitro OECD TGs to study such endocrine-related effects [i.e., (anti)oestrogenicity, (anti)androgenicity and steroidogenesis] are available, such as: OECD TG 455 (OECD 2016h), OECD TG 493 (OECD 2015g), OECD TG 458 (OECD 2020c), and OECD TG 456 (OECD 2011). Beyond methods specifically designed for the detection of these endocrine MoAs in vivo (OECD TGs 440 and 441), and reproduction/developmental studies (OECD TGs 414, 421/422, 426, 416, and 443), repeated dose toxicity studies (here summarised under “Repeated dose toxicity” section and Table 2) can also be used to assess parameters sensitive to endocrine MoAs. Existing gaps and weaknesses in current test methods for the evaluation of EDs have been discussed in 2017 during a European expert workshop, the results of which were published in a 2018 report (EC 2018b).

One of the activities undertaken by EURL ECVAM in this context is the revision of OECD TG 458 (OECD 2020c) to include several Androgen Receptor Transactivation Assays (ARTAs). This TG is based on validated ARTAs: AR-EcoScreen (OECD 2015a), AR-CALUX (EC 2017b), or the ARTA based on 22Rv1/MMTV cell line (Sun et al. 2016).

Several screening approaches have been proposed in recent years to improve the regulatory assessment of chemicals for possible ED effects. A screening approach to prioritise substances for regulatory evaluation has been developed by ECHA, and it includes screening for potential ED properties (ECHA 2019). It is envisioned that rather than individual assays, a combination of assays (test battery) or a tiered screening strategy, including a WoE evaluation, may be more useful, as commented also by Paul Friedman and co-authors with regard to a possible screening approach to identify thyroperoxidase inhibitors (Paul Friedman et al. 2016).

Importantly, to date there are no specific OECD TGs addressing thyroid toxicity in vitro. With regard to thyroid disruptors and strategies to better assess chemicals for their thyroid signalling disrupting effects, the OECD has generated a Detailed Review Paper (OECD 2006), and has compiled a detailed scoping document summarising available in vitro and ex vivo methods suitable for the identification of thyroid disruptors (OECD 2014b). In March 2017, DG Environment and ANSES (the French Agency for Food, Environmental and Occupational Health and Safety) held a Thyroid Disruptor workshop (EC 2017e) with the goal to address and discuss interpretations of experimental data (i.e., laboratory studies, wildlife field data and human epidemiological data) in relation to the identification of thyroid disruptors, and to identify ways forward in addressing potential gaps in test methods.

In 2017, EURL ECVAM launched a call to the members of the European Union Network of Laboratories for the Validation of Alternative Methods (EU-NETVAL) (https://ec.europa.eu/jrc/en/eurl/ecvam/alternative-methods-toxicity-testing/eu-netval) for participation in a validation study with a selected number of in vitro methods suitable to measure thyroid disruptors (EC 2017c). The final aim of this validation activity is to attain a set of methods suitable to cover the known targets of thyroid disruption and that could in the future be included in OECD TGs.

Moreover, at the end of 2017, a call for tender was launched by DG Environment for the development of a study protocol for thyroid disruptor testing in the mammalian system, with the aim to improve the identification of thyroid disruptors, by either enhancing already existing OECD TGs and/or developing a new one. In particular, the endpoints that were considered during the feasibility study were: (i) heterotopias, (ii) hormone measurements, and (iii) cortical gene expression. These endpoints may be potentially added to EOGRTS (OECD TG 443) (EC 2019c).

Additionally, the H2020-funded cluster EURION, with its eight projects running for 5 years from beginning of 2019, focuses on new and improved methods, as well as screening and testing strategies for thyroid hormone disruption, endocrine-related metabolic diseases, female reproductive effects and DNT (https://eurion-cluster.eu/). The outcome of the projects will contribute to international activities on EDs at OECD level (EC 2020c).

The provisions for identifying EDs in different pieces of EU legislation, including REACH and the Cosmetic Products Regulation, are reviewed in a recently completed Fitness Check, led by the JRC (EC 2020d). The Fitness Check identified the need to update the information requirements, particularly under REACH, to improve the possibilities to identify those substances with endocrine disrupting properties. Such an update is currently in progress, which will consider the inclusion of both in vitro and in vivo mechanistic OECD TGs that can identify endocrine activity, as well as some in vivo TGs that have been enhanced to include endocrine-related endpoints. The Fitness Check also indicated that available OECD TGs are not sufficient to cover all the different ways in which the endocrine system may be disrupted. The ongoing research projects and validation activities described above will serve to provide new methods, with broader coverage, that can be adopted as OECD TGs and serve to build testing strategies for EDs, including the use of new approach methodologies (NAMs).

## Other challenges in the current regulatory landscape and recent initiatives to tackle them

### Mixture risk assessment (MRA)

In recent years, EU regulators have been facing several other challenges, such as (and not limited to) the definition of harmonized strategies to assess risks from combined exposure to multiple chemicals (i.e., mixture risk assessment, MRA). Exposure to multiple chemicals at the same time occurs in our daily life, and while the basic science and derived knowledge of mixture toxicology have progressed over the last years, it is still a matter of debate how to implement MRA in the current regulatory framework (Bopp et al. 2018b, 2019).

General principles for mixture toxicity assessment are outlined in Fig. 1.1 of the CLP Guidance (ECHA 2017c), which show the criteria to be followed for each hazard class independently, with the exception of substances classified as carcinogenic, mutagenic, or toxic for reproduction (CMR substances), or when evaluating biodegradation and bioaccumulation properties. Also in Appendix 1 of CLP Regulation (EC 2017d), paragraphs 1.1.3. report ‘*Bridging principles for the classification of mixtures where test data are not available for the complete mixture’.* Similar principles for mixture toxicity assessment are reported in the GHS (UN-GHS 2019), which provides harmonized criteria for mixtures classification according to their health, environmental and physical hazards in the sections specific to the different endpoints. It should be considered that CLP or any other European Regulation does not require mixture toxicity testing.

At present, each chemical is subject to an individual risk assessment, whereas MRA is usually not (appropriately) considered (Tralau et al. 2015). Moreover, EU chemical regulations operate (almost exclusively) in regulatory remits (i.e., on a chemical-by-chemical basis), but this approach may not be appropriate in cases when two or more chemicals elicit the same toxic effect (Evans et al. 2016). Methodologies to characterize combined effects and the possibility to assign substances to one or several common assessment groups have been discussed in two EFSA Scientific opinions related to active substances in plant protection products (EFSA 2013a, b) and a general Guidance document (EFSA 2019). In particular, the EFSA Panel on Plant Protection Products and their Residues (PPR) suggested that MRA could be assessed starting from the concept of dose addition for both, chemicals acting through similar MoA and those acting through dissimilar MoA, when leading to the same adverse effect (EFSA 2013b).

The most recent consolidated version of CLP (EC 2017d) provides classification criteria for mixtures for the different endpoints considered above, providing bridging principles when data are not available for the complete mixture, or are available only for some components of the mixture. Also dose addition-based concepts are suggested. Under REACH, combinations of chemicals are only addressed for multi-constituent substances (MCS) and substances of unknown or variable composition, complex reaction products or of biological origin (UVCBs). However, four phthalates were restricted under REACH on the basis of a risk assessment considering their combined exposure and results from monitoring studies with a limit value referring to their combined un-intentional exposures (ECHA 2017a).

With regards to cosmetic ingredients, usually they are assessed individually and in combinations in the composition of the final products. When data are available from industry or from European Agencies, other products than cosmetics, including the same ingredients, are also considered in the assessment.

To facilitate MRA, it has been shown how mechanistic information derived using twenty-first century methods in combination with AOPs and networks of AOPs (see also “Strategic and conceptual frameworks to integrate alternative methods in current EU regulatory context” section) could support and enable assessing mixtures in component-based and whole-mixture approaches (Bopp et al. 2018b, 2019).

### Implementing the 3Rs in current regulatory testing paradigm

Strategies to integrate up-to-date in vitro and in silico methods and models in existing or new regulatory testing strategies have been discussed at the European and international level, and efforts to develop harmonized recommendations to ensure worldwide acceptance of alternative methods and strategies have been globally undertaken (e.g., with the ICATM initiative). At the European level, Directive 2010/63/EU (EU 2010) on the protection of animals used for scientific purposes includes a number of duties (Article 48 and Annex VII) to foster the 3Rs. Additionally, several pieces of EU Regulations, such as REACH (EC 2020g) and the Cosmetic Products Regulation (EC 2020e) and their amendments have contributed to the implementation of the 3Rs, by referring to, and encouraging the use of, alternatives to animal testing. More recently, the Community Strategies on combined exposures (Bopp et al. 2015, 2018a; Kienzler et al. 2016) and on EDs (Bopp et al. 2017; Munn et al. 2016) support the use of non-animal methods for safety assessment.

**Fig. 1:**
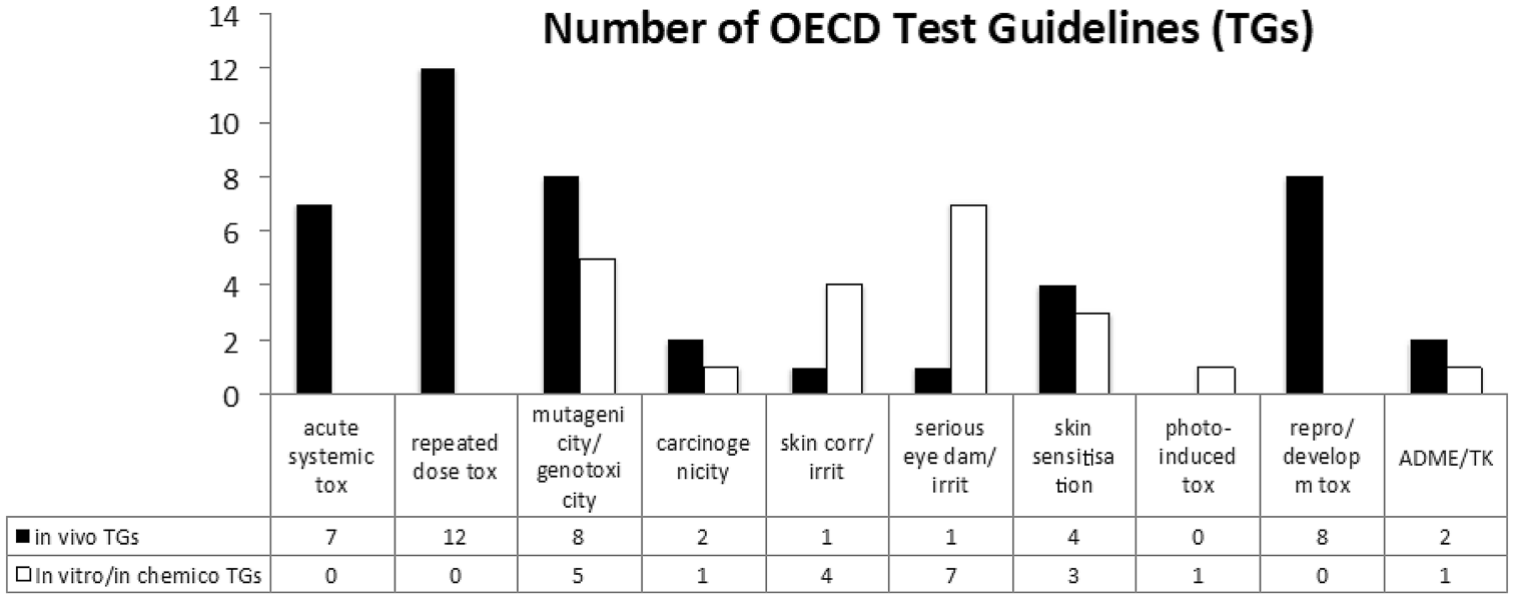
Bar graph summarising the numbers of available OECD Test Guidelines (TGs) addressing the assessment of the human health-related endpoints here described, comparing in vivo TGs (black bars) and in vitro*/*in chemico TGs (white bars)

Since the publication of such regulations and GDs, much progress has been made with the promotion, implementation and validation of alternatives to animal testing. This is reflected by the fact that, for some specific endpoints, chemicals are often tested using non-animal approaches, for example in the case of skin corrosion and irritation and serious eye damage/irritation (with globally 11 in vitro OECD TGs), skin sensitisation (with 3 available in vitro*/*in chemico OECD TGs), and mutagenicity/genotoxicity (with 5 available in vitro OECD TGs) (Fig. 1, white bars). Notwithstanding, chemical evaluation still heavily relies on the use of animals (mainly rodents), in particular for acute systemic toxicity, repeated dose toxicity and reproductive and developmental toxicity (Fig. 1, black bars).

With regards to the number of animal used for scientific purposes, Directive 2010/63/EU has put in place a more comprehensive reporting framework for Member States, and in February 2020, more precise estimates of animal use in Europe during the years 2015 to 2017 have been made available. In this report, mice, fish, rats and birds, together represent over 92% of the total numbers of animals used for scientific purposes, with most uses being in basic research (45%), followed by translational/applied research (23%) and regulatory use (23%) (EC 2020a). Notably, the report also expresses concern with the uses of animals in areas where alternative methods have already reached regulatory acceptance (such as in the areas of skin irritation/corrosion, serious eye damage/eye irritation, and pyrogenicity testing) (EC 2020a).

Remarkably, as commented in the ECHA’s fourth report on the use of alternative methods to animal testing under REACH (ECHA 2020), read-across is becoming the most commonly used adaptation, which has led to a reduction of experimental studies; additionally, the use of in vitro and in chemico non-animal test methods has tripled for skin corrosion/irritation, quadrupled for serious eye damage/eye irritation and increased by more than 20-fold for skin sensitisation.

## Strategic and conceptual frameworks to integrate alternative methods in current EU regulatory context

The development of alternative test methods based on the use of human cells and tissue cultures (from monolayer cell (co)cultures, to organotypic three-dimensional (3D) cell models, microfluidics organ-on-chip systems, 3D- and 4D-bioprinting, etc.), multiple highthroughput ‘omics’ technologies, and computational analytical methods (e.g., IVIVE, PBK, and pharmacodynamics), may in the future contribute to reduce the number of animals used in both biomedical research and regulatory toxicology.

While the application of such individual approaches may not be suitable to adequately mimic complex physiological and toxicological endpoints, the integration of in vitro (and in silico) methods may mimic certain aspects of biological complexity, to enable the prediction of certain human health effects ideally better than animal studies. It is considered that the use of human-derived cells and tissues, coupled with microphysiological system approaches (Marx et al. 2016), will increase the predictive capacity of toxicological effects of chemicals or new drugs to humans (Archibald et al. 2018), while enabling mechanistic understanding of how chemicals and drugs produce their effects (Dehne et al. 2017; Tralau et al. 2012; Wobus and Loser 2011).

As described in “Implementing the 3Rs in current regulatory testing paradigm” section, some of the current OECD TGs are based on the use of alternative approaches (Fig. 1), supporting the 3Rs. Also, waiving principles are in place to reduce the number of animals, and after the marketing ban of cosmetics tested on animals in 2013, testing of cosmetic ingredients is no longer possible under the Cosmetic Products Regulation, and this has triggered the development of new approaches based on non-animal methods and models (SCCS 2018). Nevertheless, regulators generally have traditionally adopted a cautious approach when discussing the possibility to phase out traditional animal approaches in favour of alternative methods, which has been justified on the basis of the need to treat human safety as paramount (Tralau et al. 2012). One of the major arguments in favour of this precautionary attitude is the fact that alternative methods may be integrated in current regulatory testing approaches only upon their international acceptance and validation. Nevertheless, it is worth noticing that most in vivo methods have never been formally validated (Tralau et al. 2015).

In vitro methods may also allow elucidating how inter-species differences can have an impact on chemical response, as shown for instance in Baumann et al. study, where differences in chemical effects on neurodevelopmental key events were described comparing human and rat neurospheres (Baumann et al. 2016). Several studies have highlighted species-specific differences, e.g., in the pace of development (Rayon et al. 2020), in liver cytochrome P450 and transport protein (Hammer et al. 2021), in the metabolic capacity and clearance of liver microsomes (Ma et al. 2017), in the expression of GABA-A receptor in T lymphocytes (Mendu et al. 2012), in the expression of nociceptive markers and ion channels between human and mouse iPSC-derived nociceptors (Schoepf et al. 2020). Altogether, this underlines the importance to test chemical effects on human toxicological endpoints using human-relevant test systems. It should also be considered that the inherent limitations of in vitro testing should be accepted in the same way as in vivo testing limitations are currently accepted (Tralau et al. 2012). An approach to systematically describe the uncertainties and complexity of the standard animal testing and assessment approach on the example of carcinogenicity has been explored by Paparella et al. (Paparella et al. 2017).

In the last decade, several strategies have been undertaken by different organizations and institutions, such as EURL ECVAM (EC 2017b, 2018a), to promote the development and the dissemination of alternative methods and approaches, encouraging the assessment of chemicals without relying on animal testing, covering different regulatory areas and their related needs. In this context, the AOP conceptual framework is currently considered as a relevant instrument in toxicology, as it allows portraying existing knowledge concerning the association between a molecular initiating event (MIE) and an adverse outcome (AO) in a chemical-agnostic way at different levels of biological complexity that are relevant to risk assessment (i.e., any chemical perturbing the MIE with sufficient potency and duration is likely to trigger that AOP) (Leist et al. 2017). The process of developing AOPs is nowadays well defined and efforts have been made to support broad and international participation through training and outreach (Edwards et al. 2016). This ‘mode of action’ framework further enables the development of IATA, which represents a science-based pragmatic approach suitable for the characterisation of chemical hazard. Such approaches rely on an integrated analysis of existing information, together with the generation of new information using testing strategies (OECD 2020a). IATA, by following an iterative method, are meant to answer a defined question in a specific regulatory context, accounting for the uncertainty associated with the decision context, and can include results of assays at various levels of biological complexity, such as in silico, (Q)SAR, read-across, in chemico, in vitro, ex vivo, in vivo, omics technologies, and AOPs (Edwards et al. 2016).

AOP-driven IATA could facilitate regulatory decision regarding potential hazards, and the risk and/or the need for further targeted testing. To define the safe and unsafe concentrations for risk assessment, potency information would be needed, and some IATA (e.g., for skin sensitisation) might be able to account for these aspects.

IATA for skin irritation/corrosion, serious eye damage/eye irritation and skin sensitisation are discussed in the OECD GDs 203 (OECD 2014a), 263 (OECD 2017b), and 256 (OECD 2016c), respectively. Such IATA include three parts: (i) retrieving and gathering of existing information, (ii) WoE analysis on all collected information, and, if no conclusion can be drawn, (iii) generation of new testing data. In particular, given the complexity of the skin sensitisation pathway, a one-to-one replacement of animal testing with a single non-animal method has not been attained so far, and instead a combination of different assays to capture different KEs of this AOP (Covalent Protein binding leading to Skin Sensitisation) (Landesmann and Dumont 2012; OECD 2012) represents a more reliable approach. For this specific endpoint (skin sensitisation), various in vitro assays have been formally validated and adopted at the regulatory level (Table 2): the direct peptide reactivity assay (DPRA) and Amino acid Derivative Reactivity Assay (ADRA) [TG 442C (OECD 2020b)], the KeratinoSens™ and LuSens assays [TG 442D (OECD 2018j)] and assays addressing the activation of dendritic cells (h-CLAT, U-SENS™ and IL-8 Luc test methods) included in TG 442E (OECD 2018k). Along this line, a number of Defined Approaches (DAs) integrating information from multiple non-animal methods (e.g., in silico, in chemico, in vitro) and other relevant information (e.g., physico-chemical properties) have been developed for the purpose of skin sensitisation hazard assessment and/or potency categorisation. The OECD GD 255 (OECD 2016d) provides principles and templates for reporting DAs to testing and assessment that can be used as either stand-alone or one of the components within IATA.

In the context of IATA, the OECD GD 275 (OECD 2017a) describes four IATA case studies as examples of predictions that are fit for regulatory use, relying specifically on alternative methods and taking into account exposure considerations and kinetics.

The OECD Project 4.116 added to the OECD Test Guidelines workplan in 2017 and led by EURL ECVAM, ICCVAM and Health Canada, aims to develop a Guideline on DAs for Skin Sensitisation. Following a special meeting of the Working Group of National Coordinators of the Test Guideline programme (WNT) in December 2017, an Expert Group on DAs for Skin Sensitisation (DASS), was convened in early 2018. Through face-to-face meetings, teleconferences and written commenting, the Expert Group provided input on a framework for evaluating DAs, and has applied the evaluation criteria to a first set of relatively simple, rule-based DAs based on OECD adopted in chemico and in vitro test methods. These DAs are under consideration for inclusion in a draft Guideline that aims to substitute the animal tests.

Moreover, several competitive research projects, such as SEURAT-1 (www.seurat-1.eu), EU-ToxRisk (www.eu-toxrisk.eu), and EuroMix (www.euromixproject.eu) have been launched in recent years in Europe, with the main goal to promote the use of alternative methods and progress towards an animal-free toxicological assessment. In particular, EU-ToxRisk, a continuation of the prior FP7 research initiative SEURAT-1, integrates advancements in cell biology, ‘omics’ technologies, systems biology and computational modelling to increase mechanistic understanding of cause-consequence relationships of chemical adverse effects. EuroMix specifically aims at developing an experimental tiered strategy for the risk assessment of mixtures of chemicals derived from multiple sources, taking into account prioritisation criteria for chemicals based on their exposure and hazard characteristics, and evaluating the role of MoA in grouping chemicals into cumulative assessment groups.

Along the same line, EDC-MixRisk (http://edcmixrisk.ki.se/) integrates epidemiology and experimental biology to improve risk assessment of exposure to mixtures of EDs. Another project, HBM4EU—The European Human Biomonitoring Initiative (www.hbm4eu.eu) aims at coordinating and advancing human biomonitoring in Europe, providing better evidence of the correlations between chemical exposure and possible health effects, and supporting policy-making.

Finally, following an OECD mandate, EURL ECVAM has drafted a guidance document on Good In Vitro Method Practices (GIVIMP) (OECD 2018a), taking into account good scientific, technical and quality practices aimed at ensuring that in vitro method development and implementation for regulatory use become more efficient and effective. Altogether, these projects and initiatives may help bridge current gaps in regulatory testing, and facilitate a paradigm shift towards a mechanistically driven hazard identification, characterization and risk assessment.

## Discussion

Understanding current regulatory requirements for the assessment of chemical and cosmetic ingredient effects on human health is essential to identify possible knowledge gaps, and evaluate how alternative methods could be better integrated in current regulatory landscape. Along this line, EU regulations call for the use of alternative non-animal methods, and over the last decade, an increasing number of alternative approaches has been developed and formally adopted. These methods have increased mechanistic understanding of toxicological effects, contributing to better hazard identification and risk assessment. However, several issues still need to be faced, such as the need to (i) better characterize toxicity pathways, (ii) develop assays suitable to bridge currently uncovered scientific gaps, (iii) increase our understanding of the links between in vitro readouts and the (adverse) outcomes in target species, (iv) better define applicability domains for alternative methods, and (v) foster the broad and harmonized implementation of currently available alternative methods. These were recognized as major challenges by different stakeholders participating in a EPAA (European Partnership for Alternative Approaches to Animal Testing) meeting organized in 2016 (Dal Negro et al. 2018).

Notably, regulatory requirements for the safety assessment of industrial chemicals and cosmetic products differ, as described in this document. To tackle complex and systemic toxicity effects, integration of available information on relevant endpoints, encompassing data derived from traditional and alternative toxicology test systems, together with most recent data streams and epidemiology data sources, should be considered, as it has been recently discussed in the context of carcinogenicity testing (Corvi et al. 2017; Madia et al. 2019). Sharing of data and international cooperation among governmental bodies, as the one fostered by the ICATM initiative, are essential to improve the capacity to solve complex problems, as commented in the “OECD Regulatory Policy Outlook 2018” (OECD 2018b).

With the advancement of new technologies and models in bioscience developed by academia and industry, dialogue and knowledge sharing should span beyond the regulatory testing arena. Along this line, a recent EURL ECVAM initiative, called BEAMS (BridgE Across Methods in bioSciences) (EC 2018a), aimed at supporting greater connectivity between biosciences, and understanding how knowledge sharing and meaningful cross-disciplinarity can play a role and what form it should take.

Efficacy and predictive capacity of currently available in vivo TGs are intensively debated and generally questioned in relation to their applicability to humans (species extrapolation) as well as their sensitivity to pick up effects. It is generally perceived that a one-to-one replacement of an in vivo TG or method with an in vitro (non-animal) one is not a suitable way forward, and that biological complexity may better be mimicked by a combination of in vitro and in silico tests, following the IATA framework. Such integrated testing should in principle be able to predict human health effects better than animal studies (Archibald et al. 2018; Hartung 2009; Marx et al. 2016), helping to unravel the molecular and cellular mechanisms underlying the effects of chemicals, cosmetic products (and drugs) on human health (Dehne et al. 2017; Tralau et al. 2012; Wobus and Loser 2011). Beside the technical debate, *‘relying on data from alternatives also needs a change in mind-set, from a box ticking exercise into a fit for purpose hypothesis-driven strategy for generating relevant data*’, as emphasized in the Cosmetics Europe annual conference 2018 report (Europe 2018). A check-list approach based on in vivo TGs does not efficiently meet legislative mandates that require increased numbers of chemical assessments without a parallel increase in the use of animals and resources. These new approaches are necessary to close the gap between the number of chemicals in use and the number assessed to date.

Moreover, the recently published EU Chemicals Strategy for Sustainability, aiming at a toxic-free environment under the European Green Deal (EC 2020b) calls for innovation in chemicals safety testing to reduce dependency on animal testing. The strategy highlights the importance to improve the quality, efficiency and speed of chemical hazard and risk assessments using advanced tools, methods and models, and data analysis capacities. It is becoming more and more evident that traditional animal testing approaches simply do not match the current needs anymore. The strategy can only be effective if the paradigm-shift in toxicity testing, advocated 15 years ago, is finally becoming fully functional.

Notably, current information requirements are essentially based on apical adverse effect endpoints observed in animal tests. The current approach to replacing such tests attempts to directly relate and match mechanistic data obtained with new technologies and models with apical effects; on the other hand, a better approach might be to revise the information requirements on the basis of new ways of describing toxicity hazard to better exploit these new data streams. Indeed, it is at the moment very difficult, if not impossible, to classify a chemical on the basis of mechanistic data within the framework of current GHS and CLP criteria, which are currently based on animal studies. Work has recently started at UN level to revise the GHS criteria with a view to include in vitro*, *in silico and *in chemico* methods, as well as grouping and read across, as a basis for hazard assessment, with the ultimate goal to adapt the criteria to non-animal data.

With the increasing interconnectedness of economies and global communication, the discussion about the use of non-animal methods has clearly expanded beyond the scientific and regulatory remits, and concerns regarding the use of animals for scientific and regulatory purposes have been globally raised by the general public. A 2014 USA poll, aimed at exploring public attitudes toward the use of animals for scientific purposes, highlighted that about 47% of interviewed participants were in favour of the practice, while about 50% opposed it, with a trend towards a decreased support for animal research since 2009 (Center 2015; Sullivan 2016). Similar polls have been carried out to depict Europeans’ view on this matter, with analogous results (Clemence and Leaman 2016; EC 2010).

It is noteworthy that a trend towards the 'democratization of science' has been observed, and it is therefore becoming progressively important to understand public attitudes toward current scientific practice, and engage the society on such issues (Ormandy and Schuppli 2014). The recent European Citizens' Initiative (ECI) *Stop Vivisection* (http://www.stopvivisection.eu/), which demanded an abrogation of Directive 2010/63/EU on the protection of animals used for scientific purposes and a full replacement of animal tests with alternative methods (Menache 2016), should be proactively taken by regulators and the scientific community as an opportunity to develop new ways to engage the public on such issues, expanding the boundaries in the debate on the use of animals for scientific purposes. Again, dialogue with all stakeholders and knowledge sharing are pivotal to advance towards the goal of phasing out animal testing, as commented in the EC reply to ECI *Stop Vivisection* (EC 2015b). Recent EC initiatives are working towards this direction; in particular, EURL ECVAM had undertaken a review to map 3Rs knowledge, determine how knowledge is shared, and identify opportunities to improve on the current situation (Holley et al. 2016).

Importantly, the acceptance and use of alternative methods also require careful monitoring and appraisal by the Competent Authorities. In this regard, the European Coalition to End Animal Experiments (ECEAE), grouping about 20 animal protection organisations across the EU (https://www.eceae.org/), carried out an independent analysis of the publicly available national reports on animals used for scientific purposes (EC 2019a) (Taylor and Rego 2016). This analysis highlighted four specific regulatory tests recorded in these statistical reports, i.e., (i) skin irritation (as typically using rabbits), (ii) eye irritation (as exclusively using rabbits), (iii) skin sensitisation (as typically using mice or Guinea pigs), and (iv) pyrogenicity tests (as exclusively using rabbits), although these tests have accepted alternatives to their use, recognised under the EU legislation. While in recent years an increasing trend in the use of alternative methods for skin sensitisation has been observed, in areas such as skin irritation/corrosion, serious eye damage/eye irritation and pyrogenicity testing, concerns still exist with regards to animal uses, as highlighted in the most recent European statistics (EC 2020a).

Additionally, since the 2013 EU marketing ban of cosmetics tested on animals (EC 2013a), the European Parliament has further launched a resolution for a world-wide ban of animal testing for cosmetics (EP 2018), with the support of the EC. As commented by Cosmetics Europe (Europe 2018), the EU ban presents several caveats [e.g., in the case of cosmetics that are tested outside of the EU on animals and re-tested using alternative methods for the EU market, or considering that the testing and marketing bans do not apply to testing required for environmental endpoints or exposure of workers (ECHA 2014b)], which make the ban far less effective. Taking all these aspects into account, current acceptance and use of alternative (non-animal) methods and TGs should be a matter of transparent and open debate among all stakeholders.

Furthermore, the development of new methods (and subsequent validation/evaluation and uptake) mainly occurs as a consequence of increased funding and market opportunities. For example, the ban on animal testing for cosmetic ingredients and products triggered the development of new non-animal approaches within the cosmetics industry. Moreover, the pharmaceutical industry is also developing and using new in vitro methods and in silico technologies (e.g., machine learning and artificial intelligence), which have recently shown more promising than animal models to predict human responses (Freedman 2019; Yau et al. 2017).

ICATM will continue to explore the future outlook of NAMs in regulatory testing frameworks and identify opportunities and obstacles for their uptake in the respective ICATM jurisdictions in a world of growing awareness of the global interconnectedness of human and environmental health.

## Footnotes

1 Three Rs and 3Rs are used interchangeably.

2 A DA consists of a fixed data interpretation procedure (DIP) applied to data generated with a defined set of information sources.

3 “Testing carried out for cosmetics relevant endpoints on ingredients that have been specifically developed for cosmetic purposes and are exclusively used in cosmetic products would in the Commission's view always be assumed to be carried out 'in order to meet the requirements of this Directive/Regulation'” (Commission Communication COM/2013/135, Page 8).

4 https://echa.europa.eu/documents/10162/23010712/a-009-2018_decision_en.pdf/237e31c9-2801-c160-7e5b-7ce81a3b7f17.

5 https://echa.europa.eu/documents/10162/23010712/a-010-2018_decision_en.pdf/46612b84-29af-29ea-9192-b2506f33c8ce.
